# Ethanol Extracts of Fruiting Bodies of *Antrodia cinnamomea* Suppress CL1-5 Human Lung Adenocarcinoma Cells Migration by Inhibiting Matrix Metalloproteinase-2/9 through ERK, JNK, p38, and PI3K/Akt Signaling Pathways

**DOI:** 10.1155/2012/378415

**Published:** 2012-02-21

**Authors:** Ying-Yi Chen, Fon-Chang Liu, Pei-Yu Chou, Yi-Chung Chien, Wun-Shaing Wayne Chang, Guang-Jhong Huang, Chieh-Hsi Wu, Ming-Jyh Sheu

**Affiliations:** ^1^School of Pharmacy, China Medical University, 91 Hsueh-Shih Road, Taichung 404, Taiwan; ^2^Department of Pharmacy, Da Chien General Hospital, Miaoli 36052, Taiwan; ^3^Department of Life Science, National Chung Hsing University, 250 Kuo Kuang Road, Taichung 402, Taiwan; ^4^National Institute of Cancer Research, National Health Research Institutes, 35 Keyan Road, Zhunan, Miaoli 350, Taiwan; ^5^School of Chinese Pharmaceutical Sciences and Chinese Medicine Resources, China Medical University, Taichung 404, Taiwan

## Abstract

Cancer metastasis is a primary cause of cancer death. *Antrodia cinnamomea* (*A. cinnamomea*), a medicinal mushroom in Taiwan, has shown antioxidant and anticancer activities. In this study, we first observed that ethanol extract of fruiting bodies of *A. cinnamomea* (EEAC) exerted a concentration-dependent inhibitory effect on migration and motility of the highly metastatic CL1-5 cells in the absence of cytotoxicity. The results of a gelatin zymography assay showed that *A. cinnamomea* suppressed the activities of matrix metalloproteinase-(MMP-) 2 and MMP-9 in a concentration-dependent manner. Western blot results demonstrated that treatment with *A. cinnamomea* decreased the expression of MMP-9 and MMP-2; while the expression of the endogenous inhibitors of these proteins, that is, tissue inhibitors of MMP (TIMP-1 and TIMP-2) increased. Further investigation revealed that *A. cinnamomea* suppressed the phosphorylation of ERK1/2, p38, and JNK1/2. *A. cinnamomea* also suppressed the expressions of PI3K and phosphorylation of Akt. Furthermore, treatment of CL1-5 cells with inhibitors specific for PI3K (LY 294002), ERK1/2 (PD98059), JNK (SP600125), and p38 MAPK (SB203580) decreased the expression of MMP-2 and MMP-9. This is the first paper confirming the antimigration activity of this potentially beneficial mushroom against human lung adenocarcinoma CL1-5 cancer cells.

## 1. Introduction

Lung cancer has been the leading cause of cancer-related mortality worldwide. In some countries, it has become the number one cancer cause of death, accounting for more fatalities than prostate cancer, breast cancer, and colorectal cancer combined [1]. Human lung adenocarcinoma cell lines CL1-0, CL1-1, CL1-5, and CL1-5-F4 are a series of sublines with progressively invasive ability established by in vitro invasion screening. CL1-5 cells are a human lung adenocarcinoma cell line derived from the parental CL1 cells by five successive Matrigel selections. CL1-5 cells showed a 4- to 6-fold higher invasive ability than the parental cells and their production of 92-kDa MMP-9 also exhibited a drastic increase over that of their parental cells.

Metastasis is a characteristic of highly malignant cancers with poor clinical outcome. Malignant tumor progression depends upon the capacity to invade, metastasize, and promote the angiogenic host response. One critical characteristic that metastatic cancer cells have acquired is the ability to dissolve basement membranes and the extracellular matrix (ECM). This degradative process is mediated largely by matrix metalloproteinases (MMPs), which are a large family of at least 20 zinc-dependent neutral endopeptidases that together can degrade all known components of ECM [2]. MMP-9 is abundantly expressed in various malignant tumors and is postulated to play a critical role in tumor invasion and angiogenesis [3]. Thus, the inhibition of MMP activity, including MMP-9, is important for the prevention of cell invasion. CL1-5 cells, a human lung adenocarcinoma cell line, expressed an elevated level of MMP-2, MMP-9 and exhibited a highly invasive and metastatic ability [4, 5]. Meanwhile, the activity of MMPs is prone to the inhibition of endogenous tissue inhibitor of metalloproteinases (TIMPs), which are specific inhibitors of MMPs, and the imbalance between MMPs and TIMPs may contribute to degradation or deposition of ECM [6]. The mitogen-activated protein kinases (MAPKs) play an important regulatory role in cell growth, differentiation, apoptosis, and metastasis [7]. In addition, phosphatidylinositol-3-kinase/serine/threonine protein kinase (or protein kinase B) (PI3K/Akt) signal transduction pathway is involved in the development, progression, and metastasis of various tumors [8–10].

Traditionally, *Antrodia cinnamomea* (*A. cinnamomea*; also named niu-chang-chih in Chinese) has been used as a health food to prevent inflammation, hypertension, itchy skin, and liver cancer [11]. There is increasing evidence that *A. cinnamomea *possesses an extensive range of biological activities, including the liver protection [12], anti-inflammation [13], and antioxidation [14]. Some reports have suggested that extracts of mycelia and fruiting bodies of *A. cinnamomea *could be a potential chemotherapeutic agent against hepatoma, as well as prostate, breast, bladder, and lung cancer cells [15–20], the antimetastatic effect of *A. cinnamomea* in lung adenocarcinoma CL1-5 cancer cells is still unclear. In the present study, we investigated the antimetastatic effects of *A. cinnamomea* on a highly metastatic CL1-5 cell lines as well as the underlying mechanisms.

## 2. Materials and Methods

### 2.1. Chemicals

*A. cinnamomea* was kindly provided by Cosmox Biomedical Co. Ltd. (Taoyuan, Taiwan). RPMI Medium 1640, 3-(4, 5-dimethylthiazolyl-2)-2, 5-diphenyltetrazolium bromide (MTT), LY294002, SP600125, and SB203580 were obtained from Sigma Chemical Co. (St. Louis, MO, USA). PD98059 was purchased from Cell Signaling Technology (Beverly, MA, USA). Trypsin−EDTA, fetal bovine serum (FBS), and penicillin/streptomycin were from Gibco Life Technologies, Inc. (Paisley, UK). Cell culture supplies were purchased from Costar (Corning, Inc., Cypress, CA, USA). The antibody against AKT, Rac-1, MAPK/extracellular signal-regulated kinase (ERK) 1/2, c-Jun NH_2_-terminal kinase (JNK)/stress-activated protein kinase, and p38 MAPK proteins and phosphorylated proteins were purchased from Cell Signaling Technology (Beverly, MA, USA). Anti-ERK1/2, anti-PI3K, antifocal adhesion kinase (FAK), anti-p-FAK, and horseradish peroxidase-conjugated goat anti-mouse IgG antibody were purchased from Santa Cruz Biotechnology Co. (Santa Cruz, CA, USA), *β*-actin was purchased from Chemicon (Temecula, CA, USA).

### 2.2. Preparation of Ethanol Extract of Fruiting Bodies of *A. cinnamomea* (EEAC)

The fruiting body of *A. cinnamomea* was kindly provided by Cosmox Biomedical Co. LTD (Taoyuan, Taiwan) and identified by Dr. Chao-Lin Kuo (School of Chinese Pharmaceutical Sciences and Chinese Medicine Resource, Taiwan). *A. cinnamomea* was weighed about 1 kg and soaked in 10 L of 95% ethanol solution (extractive solvent) for 3 days at room temperature. The solid residue of the above soaked herbs was filtered and discarded through a Buchner funnel lined with Whatman filter paper, and the filtrate was concentrated to paste by vacuum distillation using the rotary evaporator (N-11, EYELA; Tokyo, Japan) and vacuum controller (VC-760, TAKARA; Tokyo, Japan) to maintain the desired pressure and temperature at 25°C with 40 mmHg. The various concentration of EEAC was further diluted with DMSO for the further use.

### 2.3. Cell Culture

CL1-5 cell lines were kindly provided by Dr. W.-S. Wayne Chang (National Institute of Cancer Research, National Health Research Institutes, Miaoli, Taiwan). The cells were cultured in RPMI-1640 medium (Gibco, Grand Island, NY, USA) with 10% fetal bovine serum (FBS, Gibco) at 37°C in a humidified atmosphere of 5% CO_2_.

### 2.4. Cytotoxicity Assay

The cells (1 × 10^5^ cells/mL) were cultured in 96-well plate containing RPMI-1640 supplemented with 10% FBS for 24 h to become nearly confluent. Then cells were cultured with EEAC for 24 h and 36 h. Then, the cells were washed twice with PBS and incubated with 100 *μ*L of 0.5 mg/mL MTT for 3 h at 37°C, testing for cell viability. The medium was then discarded and 50 *μ*L of dimethylsulfoxide (DMSO) was added. After 10 min incubation, absorbance at 590 nm was read by a microplate reader.

### 2.5. Wound-Healing Assay

For cell motility determination, CL1-5 cells (2 × 10^5^ cells/mL) were seeded in a 12-well tissue culture plate and grown to 80−90% confluence. After aspiration of the medium, the center of the cell monolayers was scraped with a sterile micropipette tip to create a denuded zone (gap) of constant width. Subsequently, cellular debris was washed with PBS, and CL1-5 cells were exposed to various concentrations of EEAC (0, 0.125, 0.25, 0.5, and 1.0 *μ*g/mL). Wound closure was monitored and photographed at 0 h and 36 h with a Nikon inverted microscope. To quantify the migrated cells, pictures of the initial wounded monolayers were compared with the corresponding pictures of cells at the end of the incubation. Artificial lines fitting the cutting edges were drawn on pictures of the original wounds and overlaid on the pictures of cultures after incubation. Cells that had migrated across the white lines were counted in six random fields from each triplicate treatment.

### 2.6. Cell Migration Assay

Tumor cell migration was assayed in transwell chambers (Millipore) according to the method reported by Weng et al. [6] with some modifications. Briefly, transwell chambers with 6.5 mm polycarbonate filters of 8 *μ*m pore size were used. CL1-5 cells (1 × 10^4^ cells/mL) and 0, 0.125, 0.25, 0.5, and 1.0 *μ*g/mL of EEAC were suspended in RPMI-1640 (100 *μ*L, serum free), placed in the upper transwell chamber, and incubated for 24 h at 37°C. Then, the cells on the upper surface of the filter were completely wiped away with a cotton swab, and the lower surface of the filter was fixed in methanol, stained with Giemsa, and counted under a microscope at a magnification of 200x. For each replicate, the tumor cells in 10 randomly selected fields were determined and the counts were averaged.

### 2.7. Determination of MMP-2 and MMP-9 by Zymography

MMPs in the medium released from CL1-5 cells were assayed using gelatin zymography (8% zymogram gelatin gels) according to the methods reported by Weng et al. with some modification [6]. Briefly, the culture medium was electrophoresed (80 V for 120 min) in a 8% SDS-PAGE gel containing 0.1% gelatin. The gel was then washed at room temperature in a solution containing 2.5% (v/v) Triton X-100 with two changes and subsequently transferred to a reaction buffer for enzymatic reaction containing 1% NaN_3_, 10 mM CaCl_2_ and 40 mM Tris-HCl, pH 8.0, at 37°C with shaking overnight (for 12−15 h). Finally, the MMP gel was stained for 30 min with 0.25% (w/v) Coomassie blue in 10% acetic acid (v/v) and 20% methanol (v/v) and destained in 10% acetic acid (v/v) and 20% methanol (v/v).

### 2.8. Western Blotting Analysis

Whole-cell lysates proteins (40 *μ*g of partially purified protein) were mixed with an equal volume of electrophoresis sample buffer, and the mixture was then boiled for 10 min. Then, an equal protein content of total cell lysate from control and EEAC-treated sample were resolved in 12% SDS-PAGE gels. Proteins were then transferred onto nitrocellulose membranes (Millipore, Bedford, MA, USA) by electroblotting using an electroblotting apparatus (Bio-Rad). Nonspecific binding of the membranes was blocked with phosphate-buffered saline (PBS) containing 1% (w/v) nonfat dry milk and TBS-T buffer (Tris-Buffer Saline with 0.1% (v/v) Tween 20) for more than 2 h. Membranes were washed with TBS-T buffer three times each for 10 min and then incubated with an appropriate dilution of specific primary antibodies in TBS-T buffer overnight at 4°C. The membranes were washed with TBS-T buffer and then incubated with an appropriate secondary antibody (horseradish peroxidase-conjugated, goat anti-mouse, or anti-rabbit IgG) for 1 h. After washing the membrane three times for 10 min in TBS-T buffer, the bands were visualized using ECL reagents (Millipore, Billerica, MA, USA). Band intensity on scanned films was quantified using Kodak Molecular Imaging (MI) software and expressed as relative intensity compared with control.

### 2.9. Statistical Analysis

Values are expressed as means ± SD and analyzed using one-way ANOVA followed by LSD test for comparisons of group means. All statistical analyses were performed using SPSS for Windows, version 10 (SPSS, Inc.); a *P* value <0.05 is considered statistically significant.

## 3. Results

### 3.1. Cytotoxicity of EEAC to CL1-5 Cells

**Figure 1:**
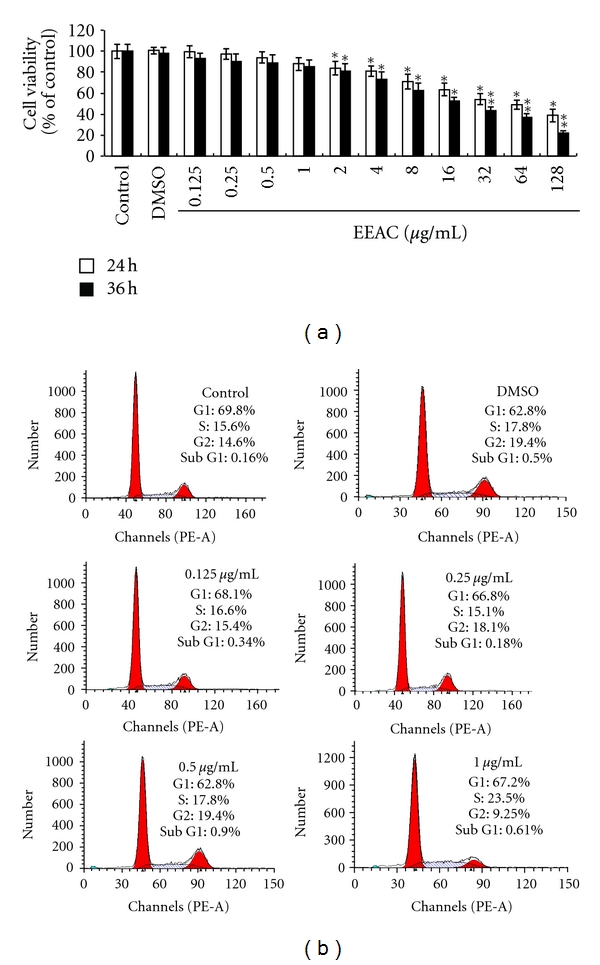
(a) Viability of CL1-5 cells incubated with EEAC (0.125, 0.25, 0.5, 1.0, 2.0, 4.0, 8.0, 16, 32, 64, and 128 *μ*g/mL) for 24 h and 36 h. Cell viability was measured using MTT assay and is expressed as % of cell survival relative to control. (b) Flow cytometric analysis of EEAC on the cell cycle of CL1-5. All the cells were treated with 1% fetal bovine serum with the addition of EEAC at 0.125 *μ*g/mL, 0.25 *μ*g/mL, 0.5 *μ*g/mL, and 1.0 *μ*g/mL for 36 h. The value on the *x*-axis represents the DNA content, while the shaded area indicates the percentage of cells at the S phase. Percentage of sub G1 contents in CL1-5 cells treated with EEAC. Values are means of three separate experiments, with standard deviation represented by vertical bars. *P < 0.05; **P < 0.01. Lower case for 24 h and upper case for 36 h.

The effect of EEAC (0−128 *μ*g/mL) on cell viability was determined by the MTT assay. After incubation for 24 h and 36 h, cell viability was not significantly affected by EEAC (0.125−1.0 *μ*g/mL), as compared to the untreated control (Figure 1(a)), indicating that EEAC is not toxic to CL1-5 cells at these concentrations. When cells were treated with EEAC at 1.0–128 *μ*g/mL for 24 h and 36 h, cell viability was significantly decreased. EEAC (0.125–1.0 *μ*g/mL) were also evaluated their apoptotic effect with flow cytometry, and SubG1 was not significantly affected by EEAC (0.125−1.0 *μ*g/mL), as compared to the untreated control (Figure 1(b)). To avoid inhibition of cell viability in the following experiments, we chose to use EEAC concentrations between 0.125 and 1.0 *μ*g/mL and an incubation time of 36 h.

### 3.2. Effect of EEAC on Wound-Healing Assay

**Figure 2:**
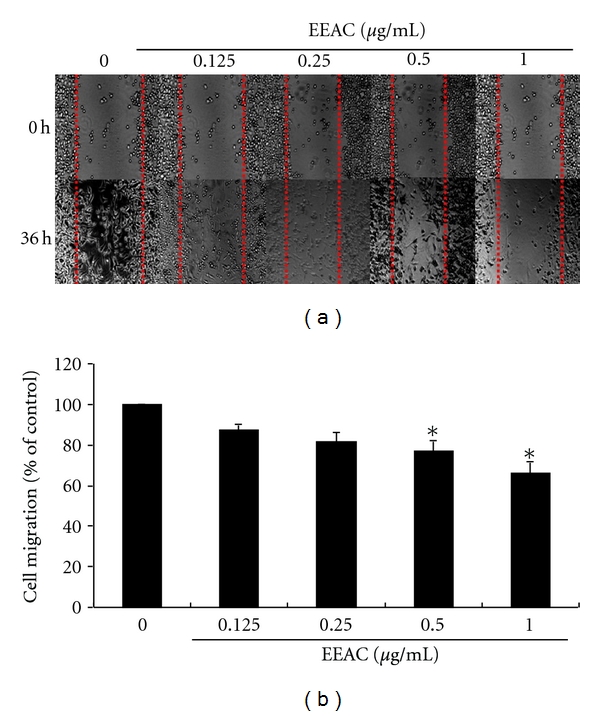
Effects of EEAC on wound healing migration of CL1-5 cells. Wound was introduced by scraping confluent cell layers with a pipet tip. CL1-5 cells were incubated with EEAC (0.125, 0.25, 0.5, and 1.0 *μ*g/mL) for 36 h, and the migration distances of cells were calculated. (a) Representative photographs of invading cells that received either control or EEAC treatment. (b) Migrated cells across the black lines were counted in six random fields from each treatment. The mean number of cells in the denuded zone is quantified by three independent experiments. Values (means ± SD, *n* = 3) differ significantly (*P* < 0.05).

We further assessed the effect of EEAC on the migration of CL1-5 cells using the wound healing assay in which the confluent monolayer was scraped with a sterile micropipette tip to create a scratch wound. As shown in Figure 2(a), EEAC inhibited the migration of CL1-5 cells in a dose-dependent manner, with 27.7% and 40.1% inhibition at 0.5 and 1.0 *μ*g/mL after incubation for 36 h, respectively, (Figure 2(b)).

### 3.3. Effect of EEAC on Migration of CL1-5 Cells In Vitro

**Figure 3:**
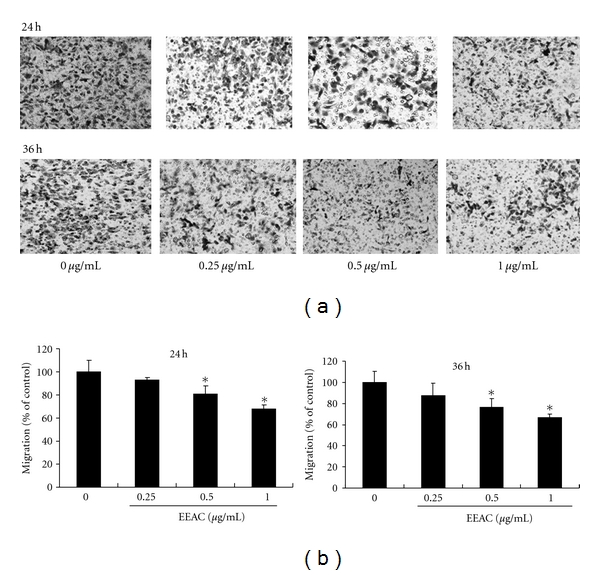
Effects of EEAC on (a) transwell migration assay of CL1-5 cells. CL1-5 cells were incubated with EEAC (0.25, 0.5 and 1.0 *μ*g/mL) for 24 h and 36 h, and the (b) transwell migration cells were calculated. Photos of the migration CL1-5 cells were taken under a microscope (200-fold magnification). Values (means ± SD, *n* = 3) differ significantly (*P* < 0.05).

The transwell assay was used to investigate the migration of CL1-5 cells at 24 h after EEAC treatment. We found that EEAC added at 0.125−1.0 *μ*g/mL significantly decreased the migration (Figures 3(a) and 3(b)) of CL1-5 cells and that these effects of EEAC were dose dependent.

### 3.4. EEAC Inhibits the Release of MMP-2 and MMP-9 in CL1-5 Cells

**Figure 4:**
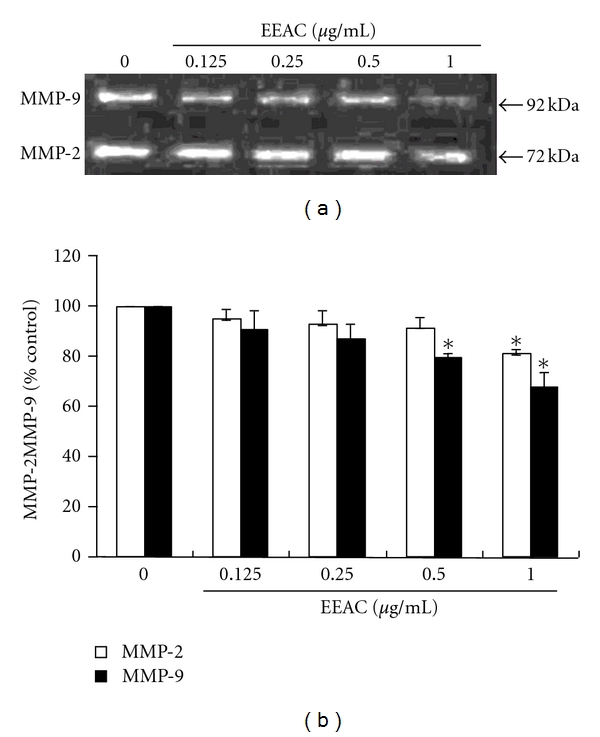
Effects of EEAC on MMP-2 and MMP-9 activities of CL1-5 cells. (a) Cells were treated with various concentrations (0.125, 0.25, 0.5, and 1.0 *μ*g/mL) of EEAC for 36 h. The conditioned media were collected, and MMP-2, and MMP-9 activities were determined by gelatin zymography. (b) The activities of these proteins were subsequently quantified by densitometric analysis. Values (means ± SD, *n* = 3) differ significantly (*P* < 0.05) (lower case for MMP-2 and upper case for MMP-9).

To examine the possible antimigration mechanisms of EEAC, we determined the activity of MMP-2 and MMP-9 in culture media of CL1-5 cells by zymographic analysis. In the absence of treatment, CL1-5 cells constitutively secreted high levels of MMP-9 and MMP-2. Our results showed that EEAC inhibited MMP-9 and MMP-2 activities in a concentration-dependent manner (Figures 4(a) and 4(b)). These results suggest that the antimigration effect of EEAC is related to the inhibition of the enzymatically degradative processes of CL1-5 migration.

### 3.5. Inhibition by EEAC of MMP-2 and MMP-9

**Figure 5:**
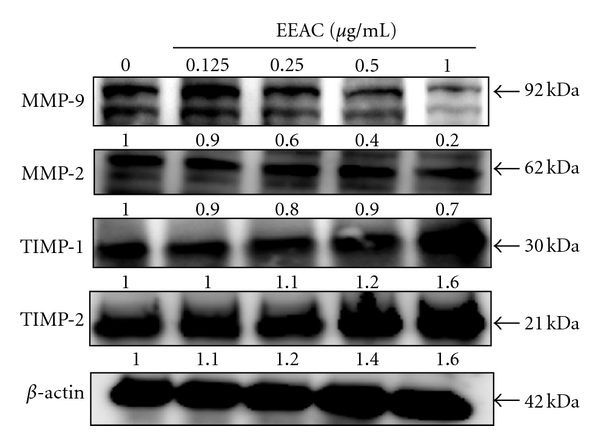
Effects of EEAC on TIMP-1, TIMP-2, MMP-9, and MMP-2 protein expression. CL1-5 cells were treated with 0.125, 0.25, 0.5, and 1.0 *μ*g/mL for 36 h, and cell lysates were subjected to SDS-PAGE followed by western blotting and subsequently quantified by densitometric analyses (using control as 100%). The values indicate the density proportion of each protein compared with control.

To examine the possible antimigration mechanisms of EEAC, we determined the MMP-2 and MMP-9 expressions in culture media of CL1-5 cells by Western blotting assay. Using Western blotting, we analyzed the effects of treating cells with various concentration of EEAC for 36 h on MMPs protein expression. Treatment of CL1-5 cells with EEAC induced noticeable reductions in MMP-9 and MMP-2 protein expression (Figure 5).

### 3.6. Effect of EEAC on TIMP-1,TIMP-2, FAK, and Phospharylated FAK (p-FAK) Expressions in CL1-5 Cells

To further explore the modulation of pro-MMP activation by EEAC, we determined TIMP-1/2 protein expression levels. As shown in Figure 5, EEAC strongly increased TIMP-1 and TIMP-2 activity in a concentration-dependent manner. To evaluate the effect of EEAC on FAK and p-FAK protein expression, CL1-5 cells were treated with EEAC at 0, 0.125, 0.25, 0.5, and 1.0 *μ*g/mL for 36 h. As shown in Figure 5, EEAC suppressed p-FAK expression in CL1-5 cells, when EEAC concentration was higher than 0.25 *μ*g/mL.

### 3.7. Effect of EEAC on PI3K, AKT, Phosphorylated AKT (pAKT), and Rac-1 Expressions in CL1-5 Cells

**Figure 6:**
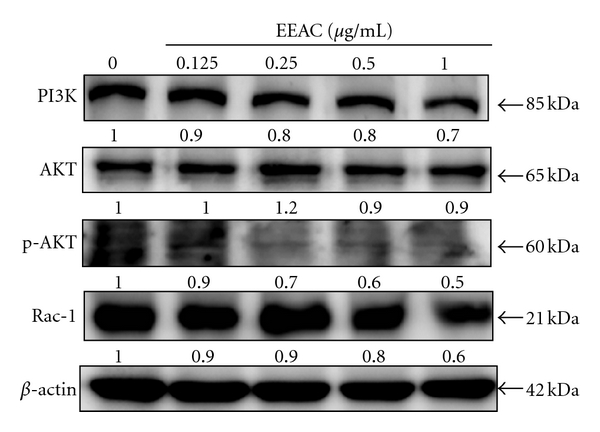
Concentration-dependent effects of EEAC on the protein expression level of PI3K, phosphorylated AKT and Rac-1. In the concentration-dependent assay, CL1-5 cells were treated with 0.125, 0.25, 0.5, and 1.0 *μ*g/mL of EEAC for 36 h. The expression of PI3K, phosphorylation of AKT and Rac-1 were analyzed by Western blotting. *β*-actin was used as a loading control. The values indicate the density proportion of each protein compared with control.

PI3Ks are a group of ubiquitously expressed lipid kinases which are important players in a major pathway of cell signaling. The PI3K/AKT pathway has been identified as a major regulator of cellular proliferation, differentiation, and death in multiple cell types [18]. To further investigate the involvement of PI3K/AKT, a series of experiments was performed to measure the expression of candidate signaling molecules upon EEAC stimulation. The results showed that incubation of CL1-5 cells with EEAC (0.125–1.0 *μ*g/mL) led to a dose-dependent decrease of PI3K and pAKT levels (Figure 6). Similarly, EEAC (0−1.0 *μ*g/mL,36 h) suppressed Rac-1 protein expression in a concentration-dependent manner, and the effect became statistically significant when EEAC concentration was higher than 0.25 *μ*g/mL (Figure 6).

### 3.8. Inhibition by EEAC of Phosphorylated FAK, ERK1/2, p38, and JNK1/2

**Figure 7:**
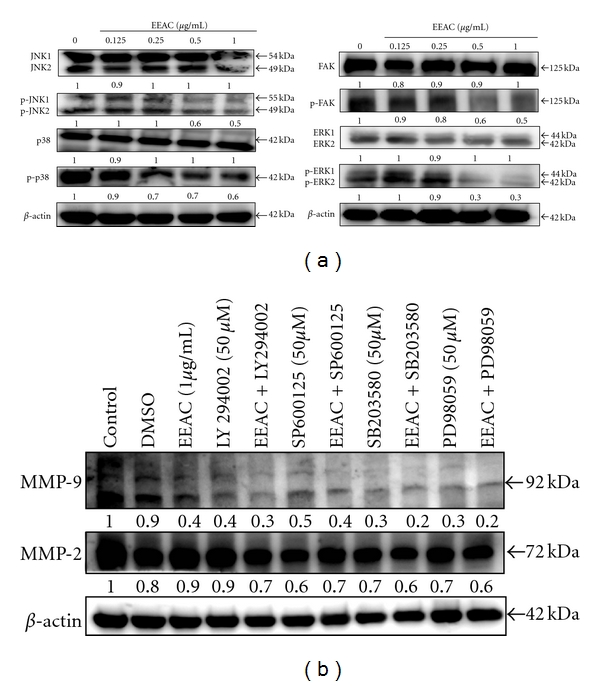
Effects of EEAC on protein levels of MAPK signaling and phosphorylated focal adhesion kinase (p-FAK) in CL1-5 cells. (a) CL1-5 were treated with several different concentrations of EEAC (0.125, 0.25, 0.5, and 1.0 *μ*g/mL) for 36 h. (b) Effects of PI3K inhibitor (LY294002; 50 *μ*M), ERK1/2 inhibitor (PD98059; 50 *μ*M), JNK inhibitor (SP600125; 50 *μ*M), and p38 MAPK inhibitor (SB203580; 50 *μ*M) on EEAC-induced protein expressions of MMP-2 and -9. Cells were pretreated with an inhibitor (50 *μ*M) at 1 h prior to the treatment with 1.0 *μ*g/mL EEAC for 36 h (total inhibitor exposure time was 37 h). Protein expressions of MMP-2 and -9 were determined by the western blotting assay.

We analyzed the phosphorylation of MAPKs in CL1-5 cells after treatment with EEAC (0.125−1.0 *μ*g/mL) for 36 h. Data in Figure 7 showed that EEAC significantly dose-dependently affect p-FAK, p-ERK1/2, p-JNK1/2, or p38 and p-p38 proteins at concentrations from 0.125–1.0 *μ*g/mL.

### 3.9. Specific Inhibitor to Analyze the Signaling Transduction

To further delineate whether the inhibition of cell migration by EEAC occurs through inhibition of PI3K, ERK, JNK, and p38 signaling, CL1-5 cells were 1 h pretreated with a PI3K inhibitor (LY294002; 50 *μ*M), ERK1/2 inhibitor (PD98059; 50 *μ*M), JNK inhibitor (SP600125; 50 *μ*M), p38 MAPK inhibitor (SB203580; 50 *μ*M), and then incubated in the presence or absence of EEAC (1.0 *μ*g/mL) for 36 h. The Western blotting assay revealed that EEAC or LY294002 (50 *μ*M), PD98256 (50 *μ*M), SP600125 (50 *μ*M), SB203580 (50 *μ*M) alone decreased MMP-9 and MMP-2 protein expressions. Furthermore, the combined treatment (50 *μ*M, LY294002, SB203580, SP600125 and PD98059 plus 1.0 *μ*g/mL EEAC) resulted in enhanced inhibition of MMP-9 and MMP-2 (Figure 7(b)).

## 4. Discussions

Several cancer cell lines including A498, NPC-TW01, Hep3B, HepG2, TW206, CL1-0, and CL1-5 have been screed for their cell cytotoxicity at 12, 24, 36, and 48 h. Our results showed that CL1-0 (IC_50_ = 16 *μ*g/mL) and CL1-5 (IC_50_ = 16 *μ*g/mL) exhibits more susceptible to EEAC than other cells for 36 h exposure. As CL1-5 cells showed higher metastatic ability, we explored the antimigration effects and mechanistic actions of EEAC in human lung adenocarcinoma CL1-5 cells. Our results demonstrate that EEAC significantly inhibited the migration (assessed using the transwell assay and the wound-healing assay) of CL1-5 cells (Figures 2 and 3). We also showed that EEAC remarkably inhibited the activities of MMP-2 and MMP-9 (Figure 4). These results demonstrated that the antimetastastic effect of EEAC was associated with the inhibition of enzymatically degradative processes of smooth muscle cells migration. To our knowledge, it is the first study to demonstrate that EEAC reduces the biochemical mechanisms of the migration in CL1-5 cells.

Several bioactive compound from the ethanol extract of *A. cinnamomea*, such as antroquinonol, 4-acetylantroquinonol B, and zhankuic acid A, have been reported to reduce cell proliferation of hepatoma, colon and leukemia cancer cells [21–23]. Our previous studies demonstrated that the marked compounds (i.e., adenosine, cordycepin, and zhankuic acid A) within EEAC were identified by HPLC and LC/MS/MS to be as indicator compounds for quality check of extraction procedure of each batch (data not shown). According to the relative concentration of external standards, the content of adenosine, cordycepin and zhankuic acid A were calculated to be 0.08, 0.16, and 235 mg/g EEAC, respectively, (data not shown). Of these, adenosine might be through binding to adenosine A3 receptors (A3-AR) to decrease the telomeric signal, resulting in G0/G1 arrest of lymphoma cell. Moreover, A3 receptor antagonist cotreated with cordycepin would reverse cordycepin-induced in mouse Leydig tumor cell death, which indicate the possible relationship between A3-AR and cordycepin-induced death effect [24, 25]. Wu et al. indicated that cordycepin could induce cell arrest at G2/M and subG1 phases followed by significant apoptotic cell death in human OEC-M1 oral cancer cell line [26]. Similarly, cordycepin has also been found to cause G2/M arrest in human bladder and colon cancer cells through upregulation of p21^WAF1^ level as well as reduction of cyclin B1, Cdc2, and Cdc25c proteins in a p53-independent manner by regulating c-Jun N-terminal kinase activation [27, 28]. Furthermore, Geethangili and Tzeng reported that zhankuic acid A exhibited cytotoxic activity against P-388 murine and leukemia cells with an IC50 value of 1.8 and 5.4 *μ*g/mL, respectively, [29].

During cancer progression, certain tumor cells become motile and attack the host tissue leading to metastasis. Focal adhesion kinase (FAK) can be activated in response to various stimuli and plays an important role in the cancer cell proliferation and metastasis [30]. Compiling evidence implies that FAK regulates focal adhesion signaling by phosphorylating various substrates and by acting as a scaffold for protein-protein interactions, as well as the MMPs-mediated matrix degradation, which consecutively regulates downstream signaling cascades [31]. FAK, a cytoplasmic kinase that is involved in ECM and integrin-mediated signaling pathways, has been suggested to have an essential role in metastasis through the modulation of tumor cell migration and invasion [32]. Majorly, integrin-mediated FAK signaling is phosphorylation of Tyr^397^. Particularly, phosphorylation of Tyr^397^ forms a high-affinity binding site for Src-homology domain 2 (SH2), domains of Src family kinase, therefore promoting Src kinase activity. As a result, a major function of FAK is to recruit and activatr Src at cell-extracellular matrix adhesion sites. Activated FAK in cancer cells relays signals through multiple downstream targets. For example, activated FAK binds the SH2 of PI3K, thereby transporting the catalytic subunit of PI3K to the membrane, where it catalyzes the phosphorylation of inositol lipids in lung cancer cell migration [33]. The residues surrounding Tyr^397^ can also constitute a sequence that binds to the Ras signaling pathway. The downstream targets of the Ras signaling pathway include ERK1/2 [34]. Indeed, these pathways are activated during integrin binding to the ECM, resulting in the transduction of external stimuli from the ECM to the nucleus [35]. Certain result suggests that anti-invasive effect in androgen-independent prostate cancer by controlling MMP-9 expression through the suppression of the EGFR/JNK pathway [36]. FAK and MAPK signaling involved in MMP-2 secretion has been shown in QG90 lung cancer cells [37]. Moreover, it was found that the MMP-9 gene promoter is partially regulated through activation of the ERK1/2 pathway [38]. In recent, p38 is implicating mediating bladder cancer invasion via regulation of MMP-2 and MMP-9 at the level of mRNA stability [39]. Also, p38*γ* MAPK activates c-Jun, and the activated c-Jun recruits p38*γ* as a cofactor into MMP-9 promoter to induce its trans-activation and cell invasion [40]. In this study, we found that EEAC inhibited the activation of FAK, as evidenced by reduced phosphorylation of FAK (Figure 7(a)). We also demonstrated that treatment with EEAC inhibited phosphorylation of ERK1/2, phospho-p38, and JNK1/2 activity (Figure 7(a)). The use of specific inhibitors revealed that PI3K, ERK, JNK, and p38 play important roles in regulating cell death induced by EEAC in CL1-5 cells (Figure 7(b)). In this study, the Western blotting assay was used to examine the effects of specific MAPK inhibitors, as the same method has often been used to examine the effects of specific MAPK inhibitors [6]. Therefore, it appears that FAK can promote CL1-5 cancer cell migration through the MAPK signaling and MMP-2 and MMP-9 pathway.

In addition, we showed that EEAC inhibited PI3K/AKT in CL1-5 cells. Thus, it seems that FAK promotes CL1-5 cancer cell migration in concert with the activation of the PI3K/AKT signaling pathways. Increased phosphorylation of FAK and its downstream targets, Src, ERK1/2, PI3K, and AKT have been shown in A549 lung epithelial cells stimulated by fibronectin. Consistent with this observation, depletion of FAK by siRNA resulted in the inhibition of Src, ERK1/2, PI3K, and AKT activity [33]. Several studies have indicated that FAK/PI3K/Akt is involved in the regulation of MMP-2 and MMP-9 activities on different cell types [9, 41]. Shibata et al. [42] have shown that stimulated ovarian cancer cells with fibronectin activated MMP-9 secretion, while both antisense oligonucleotide to FAK and dominant-negative mutation of Ras abolished this phenomenon. To assess whether EEAC inhibits the phosphorylation of FAK, AKT, and the protein level of PI3K, CL1-5 cells were treated with various concentrations of EEAC (0.125, 0.25, 0.5, and 1.0 *μ*g/mL) for 36 h. Figure 6 showed EEAC significantly inhibited the activation of FAK and AKT as shown by the decrease in the phosphorylation of FAK and AKT. In addition, EEAC inhibited the protein levels of PI3K in a dose-dependent manner (Figure 6). Zeng et al. [43] reported that activated FAK-induced PI3K is required for the production of matrix metalloproteinases (MMPs). Also, PI3K is one of the critical downstream signal molecules of FAK pathways [44]. Therefore, our results demonstrated that EEAC inhibited the expressions of p-FAK, pAKT, and PI3K.

The Rho family of small guanosine triphosphatases (GTPases) (including RhoA, RhoB, c-Raf, Rac-1, and Cdc42) are critical in regulating actin reorganization associated with cell growth, migration, transformation, and gene expression [45]. The Rho family of GTPases has been reported to be involved in the regulation of the phosphatidylinositol 3-kinase (PI3K)/AKT signaling pathway [46]. In addition, Campbell et al. [9] reported that PI3K/AKT signal transduction pathway regulates cell invasion and metastasis of nonsmall cell lung cancer (NSCLC) and is closely associated with the development and progression of various tumors. Rac1 was reported to act as a downstream effect of PI3-K in several growth factor-stimulated pathways [47] and to induce invasion and metastasis in cancer cells [48]. Rac1 activation also played a critical role in the migration of cancer cells [49]. Rac-1 has been implicated in the regulation of the contraction and retraction forces that are required for cell migration [50]. Increased Rac-1 expression enhances the phosphorylation of myosin, which cross-links actin filaments and generates contractile forces, promoting movement of the cell body and facilitating cell rear detachment. Rac-1 is also implicated in the invasion of human microvascular endothelial cells. Active-Rac-1 could induce the expression of MMP-9 metalloproteinase and promote migration of endothelial cells [51]. Rac-1 may exert this activity through upregulate transcription factors such as NF-*κ*B known to be involved in the expression of certain metalloproteinases including MMP-9. Our finding that EEAC concentration dependently decreased Rac-1 protein expression (Figure 6), suggesting that EEAC may also inhibit the metastasis of CL1-5 cells through the PI3K/Akt—Rac-1 and Rac-1—MMP-9 pathways.

**Figure 8:**
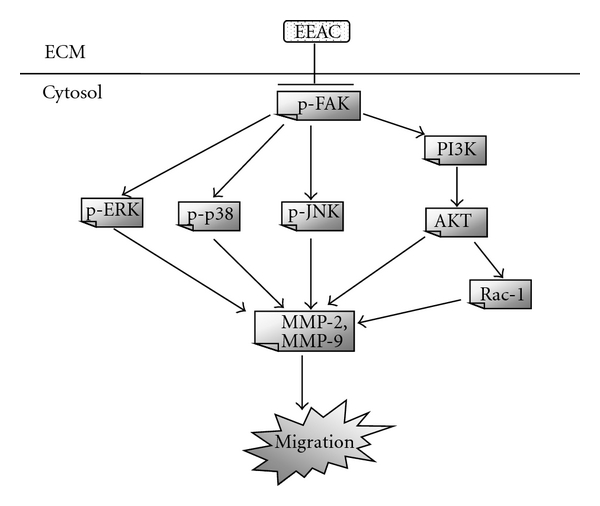
Proposed signaling pathways for EEAC-mediated inhibition against migration of CL1-5 cells. The effect of EEAC is achieved likely through the inhibition of FAK, which regulates Rac-1/MMP-9 expression through MAPK and PI3K/AKT signaling pathways.

In conclusion, we have demonstrated that EEAC inhibits the migration of human lung adenocarcinoma cancer CL1-5 cells. Mechanistically, we show that this effect of EEAC may occur through inactivation of the ERK1/2 signaling pathway, exerting inhibitory effects on FAK, p-FAK, and Rac-1 protein expressions and inhibiting PI3K, and phospho-Akt levels, thereby decreasing the activities of MMP-2, MMP-9 of CL1-5 cells (Figure 8). Further preclinical and clinical studies are required to demonstrate the potential of EEAC as an anticancer agent.

## Abbreviations

MMPs: Matrix metalloproteinases
uPA: Urokinase-type plasminogen activator
ECM: Extracellular matrix
MAPK: Mitogen-activated protein kinase
ERK: Extracellular signaling-regulating kinase
JNK/SAPK: c-Jun N-terminal kinase/stress-activated protein kinase
PI3K: Phosphoinositide 3-kinase
FAK: Focal adhesion kinase
RhoA: Ras homologue gene family, member A.
